# Retraction: Qualitative and quantitative determination of chemical constituents in Jinbei oral liquid, a modern Chinese medicine for coronavirus disease 2019, by ultra-performance liquid chromatography coupled with mass spectrometry

**DOI:** 10.3389/fchem.2025.1754927

**Published:** 2025-12-05

**Authors:** Frontiers Editorial Office

The journal retraction the February 7 2023 article cited above.

Following publication, concerns were raised regarding the scientific validity of the article. An investigation was conducted in accordance with Frontiers’ policies. The authors failed to provide a satisfactory explanation and, as a result, the conclusions of the article have been deemed unreliable, and the article has been retracted.

This retraction was approved by the Chief Editors of *Frontiers in Chemistry* and the Chief Executive Editor of Frontiers. The authors do not agree to this retraction.

